# Tolerogenic dendritic cells and TLR4/IRAK4/NF-κB signaling pathway in allergic rhinitis

**DOI:** 10.3389/fimmu.2023.1276512

**Published:** 2023-10-17

**Authors:** Chenglin Kang, Xiaomei Li, Peng Liu, Yue Liu, Yuan Niu, Xianhai Zeng, Hailiang Zhao, Jiangqi Liu, Shuqi Qiu

**Affiliations:** ^1^ Department of Graduate and Scientific Research, Zunyi Medical University Zhuhai Campus, Zhuhai, China; ^2^ Department of Otolaryngology, Longgang E.N.T Hospital and Shenzhen Key Laboratory of E.N.T, Institute of E.N.T Shenzhen, Shenzhen, China; ^3^ Department of Otolaryngology, Second People’s Hospital of Gansu Province, Lanzhou, China; ^4^ Department of Neurology, Second People’s Hospital of Gansu Province, Lanzhou, China

**Keywords:** tolerogenic dendritic cells, TLR4/IRAK4/NF-κB signaling pathway, immune response, immune tolerance, allergic rhinitis

## Abstract

Dendritic cells (DCs), central participants in the allergic immune response, can capture and present allergens leading to allergic inflammation in the immunopathogenesis of allergic rhinitis (AR). In addition to initiating antigen-specific immune responses, DCs induce tolerance and modulate immune homeostasis. As a special type of DCs, tolerogenic DCs (tolDCs) achieve immune tolerance mainly by suppressing effector T cell responses and inducing regulatory T cells (Tregs). TolDCs suppress allergic inflammation by modulating immune tolerance, thereby reducing symptoms of AR. Activation of the TLR4/IRAK4/NF-κB signaling pathway contributes to the release of inflammatory cytokines, and inhibitors of this signaling pathway induce the production of tolDCs to alleviate allergic inflammatory responses. This review focuses on the relationship between tolDCs and TLR4/IRAK4/NF-κB signaling pathway with AR.

## 1 Introduction

Dendritic cells (DCs) are bridges between innate and adaptive immune responses, they not only act as key immunomodulatory factors driving T cell initiation and activation, but also have tolerogenic functions that promote immune tolerance (1–3). Whether DCs exhibit immunogenicity or tolerance depends on their different subsets of them and the different stimuli to which they are exposed (4). Exposure of DCs to allergens leads to enhanced priming, which further mediates allergic inflammatory responses (5, 6). However, repeated low-dose exposure to allergens has the potential to make DCs tolerant (7). Tolerogenic DCs (tolDCs) are DCs with immunomodulatory functions that slow the allergic response by killing T cells (7). This would be beneficial in the treatment of AR.

DCs play an integral role in the pathogenesis and treatment of allergic rhinitis (AR). DCs recognize pathogen-associated molecular patterns (PAMPs) or lipopolysaccharide (LPS) through Toll-like receptor 4 (TLR4) expressed on their membrane to trigger signaling cascades (2, 6, 8). TLR4/nuclear factor kappa-B (NF-κB) regulates the balance of T helper type 1 (Th1) and T helper type 17 (Th17) by affecting the maturation and migration of DCs, which in turn affects the development of AR (9). The TLR4/interleukin-1 receptor-associated kinase 4 (IRAK4)/NF-κB signaling pathway eventually leads to the production of pro-inflammatory cytokines that initiate inflammatory and immune responses (10). Blocking the propagation of the TLR4/IRAK4/NF-κB signaling pathway with tolDCs may become a therapeutic target for AR.

## 2 Overview of DCs

DCs can be categorized into conventional DCs (cDCs) and plasmacytoid DCs (pDCs) according to their morphological features and function (2, 11). pCDs, primarily found in lymphoid organs, are characterized by the production of large amounts of type I interferon (IFN) after recognition of foreign nucleic acids (2, 12). In addition to producing IFN, pDCs also secrete pro-inflammatory cytokines and chemokines such as interleukin-6 (IL-6), IL-12, CXC-chemokine ligand 8 (CXCL8), CC-chemokine ligand 3 (CCL3) and more, and these chemokines attract immune cells to sites of inflammation (13).. MHC class II molecules as well as costimulatory molecules CD40, CD80, and CD86 can be expressed by pDCs, although not as efficiently as cDCs (13). cCDs consist of two main subsets: cDC1 and cDC2, which are present in almost all tissues (11, 14–16). cDC1 and cDC2 normally exert their roles in the priming of CD8 T cells and CD4 T cells, cDC1 primarily presents antigens to CD8 T cells, while cDC2 preferentially initiates various immune responses of CD4 T cells (11, 17). The locations of cDC1 and cDC2 in lymphoid and nonlymphoid organs vary, which affects their interactions with other immune cells and potentially antigens to which they are exposed (15).

Both pDCs and cDCs play important roles in immune regulation. The regulatory function of DCs depends on their activation state, which might impact their ability to induce immunity or tolerance (2, 16). The activity of DCs is closely related to the presence of immunosuppressive factors (18). Upon encountering danger signals, DCs are activated and costimulatory molecules on their surface are upregulated, followed by the production of chemokines and cytokines (1). Inflammatory cytokines and chemokines induce allergic inflammatory responses in the pathogenesis of AR. Allergens are taken up by immature DCs (imDCs) and presented to naïve T cells, which induce DCs maturation, and mature DCs (mDCs) promote adaptive immune responses by inducing effector T cells (18, 19). ImDCs induce immune tolerance by decreasing CD40 expression and increasing IL-10 expression (20). In this procedure, imDCs primarily capture allergens while mDCs mainly act as APCs (21, 22). The imDCs can produce immunogenic, pro-inflammatory mDCs as well as semi-mature DCs that have the potential to acquire tolerogenic functions (23).

**Figure 1:**
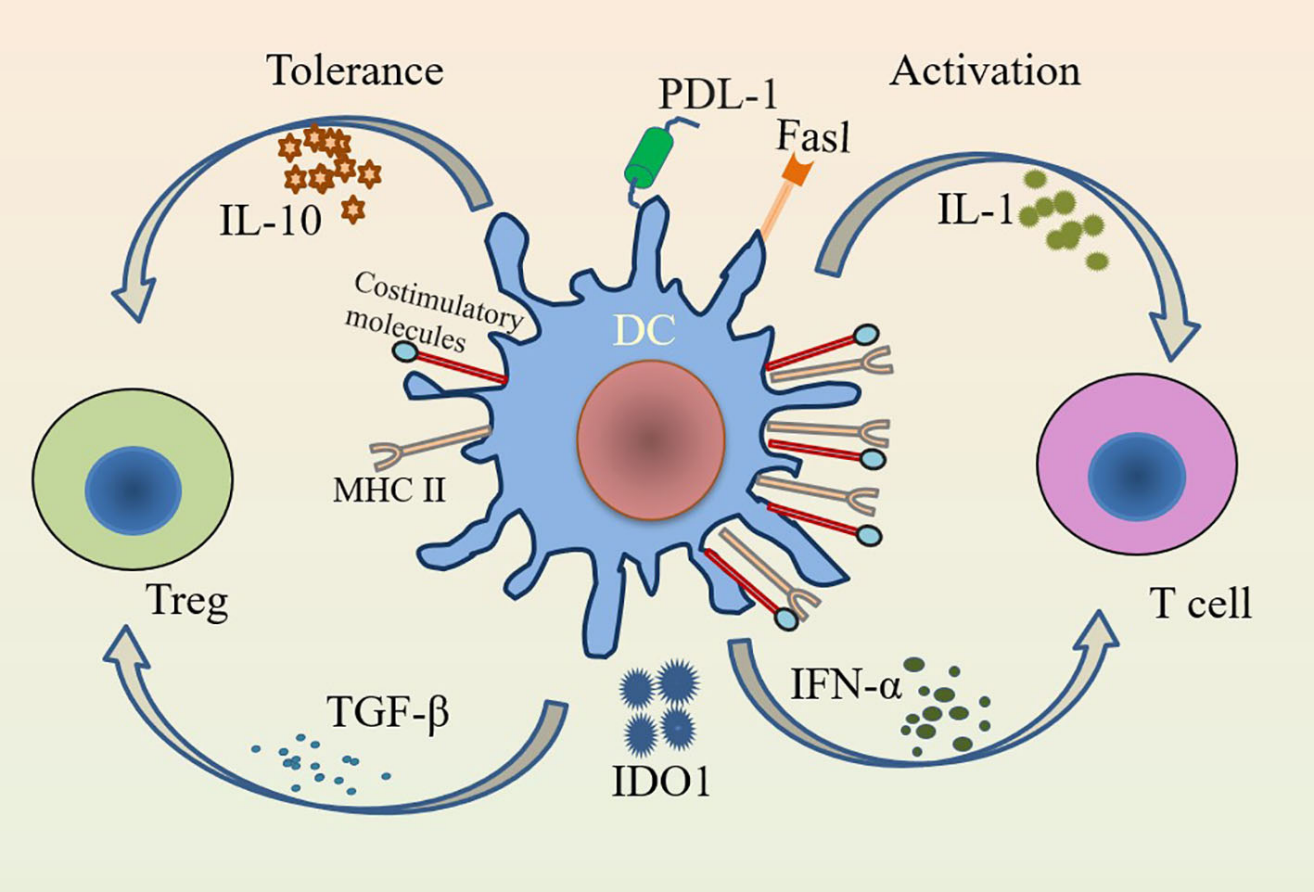
Immunogenicity and tolerance of DCs. DCs activate T cells in the presence of IL-1 and IFN-α under pro-inflammatory conditions; while DCs have tolerogenic functions that induce Tregs to suppress inflammation in the presence of inhibitory factors such as IL-10 and TGF-β. Activation of DCs highly expresses MHC class II and costimulatory molecules (CD80/CD86 and CD40), while tolerance of DCs lowly expresses them.

In addition to participating in the immune response, DCs are important modulators of central and peripheral tolerance (24). The inability of newly formed T and B lymphocytes to respond to self-antigens is known as central tolerance (25). DCs maintain central tolerance by regulating the negative selection of self-antigens (26). Increased synthesis of indoleamine-2,3-dioxygenase (IDO)1, upregulated expression of FasL, and induction and maintenance of T cell incompetence by programmed cell death ligand 1 (PDL-1) can promote peripheral tolerance in DCs (26, 27). In contrast to peripheral tolerance, the function of DCs in central tolerance appears to be very limited and may be limited to promoting tolerance to a small subset of self-antigens (28). The induction and maintenance of immune tolerance in DCs is critical for the development of inflammatory diseases. IL-10 and TGF-β have strong anti-inflammatory effects and can induce tolerance of DCs, while IL-1 and IFN-α have obvious pro-inflammatory effects and can promote the activation of DCs (**Figure 1**) (29). The presence or absence of pro-inflammatory cytokines seems to be decisive in the induction of immunity or tolerance, respectively (4). Both cDCs and pDCs have distinct roles in inducing immune tolerance (30). pDCs maintain immune tolerance by secreting IL-10 and other immunosuppressive mediators, inducing regulatory T cells (Tregs) and inhibiting the secretion of Th2 cells (12, 31). cDCs can induce immune tolerance by initiating T cells and can also lead to peripheral tolerance by inducing T cell incompetence or deletion (30). IDO1 also helps to maintain the tolerance of cDCs (16, 27). The migration pathways and functions of pDCs and cDCs differ due to differences in the expression of chemokines and chemokine receptors (9). The functions of different subtypes of DCs vary and are regulated by environmental factors, and changes in the external environment have an impact on the balance between their tolerance and immunity (32).

## 3 TolDCs

TolDCs are generally regarded as a type of steady-state semi-mature DCs, including most imDCs and some cells with advanced maturation states (23, 33, 34). With the ability to re-establish immune tolerance, tolDCs are a specific subset of DCs that overexpress tolerance markers and release tolerance cytokines while underexpressing T cells and costimulatory molecules (35, 36). They can be subdivided into induced tolerogenic DCs (itDCs) and natural tolerogenic DCs (ntDCs) (9, 37). DCs that promote immune responses in response to some inducing signals (agonists, physiological conditions, drugs) to acquire tolerogenic functions are known as itDCs, and ntDCs refer to DCs that inherently promote T cell tolerance (including T cell anergy, T cell depletion, and peripheral Treg cells transformation) in the absence of specific extrinsic signals (37). ItDCs favor the maintenance of homeostasis under pro-inflammatory conditions, while ntDCs contribute to the establishment of tolerance under homeostatic conditions (9). ItDCs and ntDCs are not separate populations, they overlap and may collaborate within organizations (23). Modulation of DC-induced tolerance can influence the immune response, both for itDCs and ntDCs (37).

TolDCs promote tolerance by facilitating the induction and expansion of different subsets of regulatory lymphocytes (38). They can acquire tolerogenic functions by inducing Tregs proliferation, autoreactive T cell incompetence and apoptosis, and interact with naïve T cells to promote tolerance (4, 39, 40). Treg cells or allergen-specific type 1 regulatory T (Tr1) cells also induce tolDCs to regulate immune responses (41). Both tolDCs and imDCs induce Tregs, but the former are more stable and do not produce pro-inflammatory cytokines (33). With plasticity functions, tolDCs play important roles in maintaining homeostasis and regulating inflammation (39). Inhibitory receptor signaling is one of the essential factors for tolDCs to suppress pro-inflammatory immune responses and induce immune tolerance (42). Immunosuppressive cytokines secreted by them can induce differentiation of Tregs to further mediate tolerogenic immune responses (24, 43). TGF-β, a cytokine with immunosuppressive functions, regulates the function of tolDCs by promoting Tregs expansion and impairing the differentiation, activation and proliferation of CD4 and CD8 T cells (44). IL-10 downregulates DC expression of MHC class II and costimulatory molecules and reverses the effects of pro-inflammatory cytokines, and IL-10-induced tolDCs release higher levels of IL-10 (37, 45). IL-10 also upregulates FasL and PDL-1 expression on tolDCs and reduces inflammatory responses by inhibiting NF-κB (34, 44). TGF-β/IL-10 signaling plays a key role in tolDCs-mediated Tregs amplification (46, 47).

**Figure 2:**
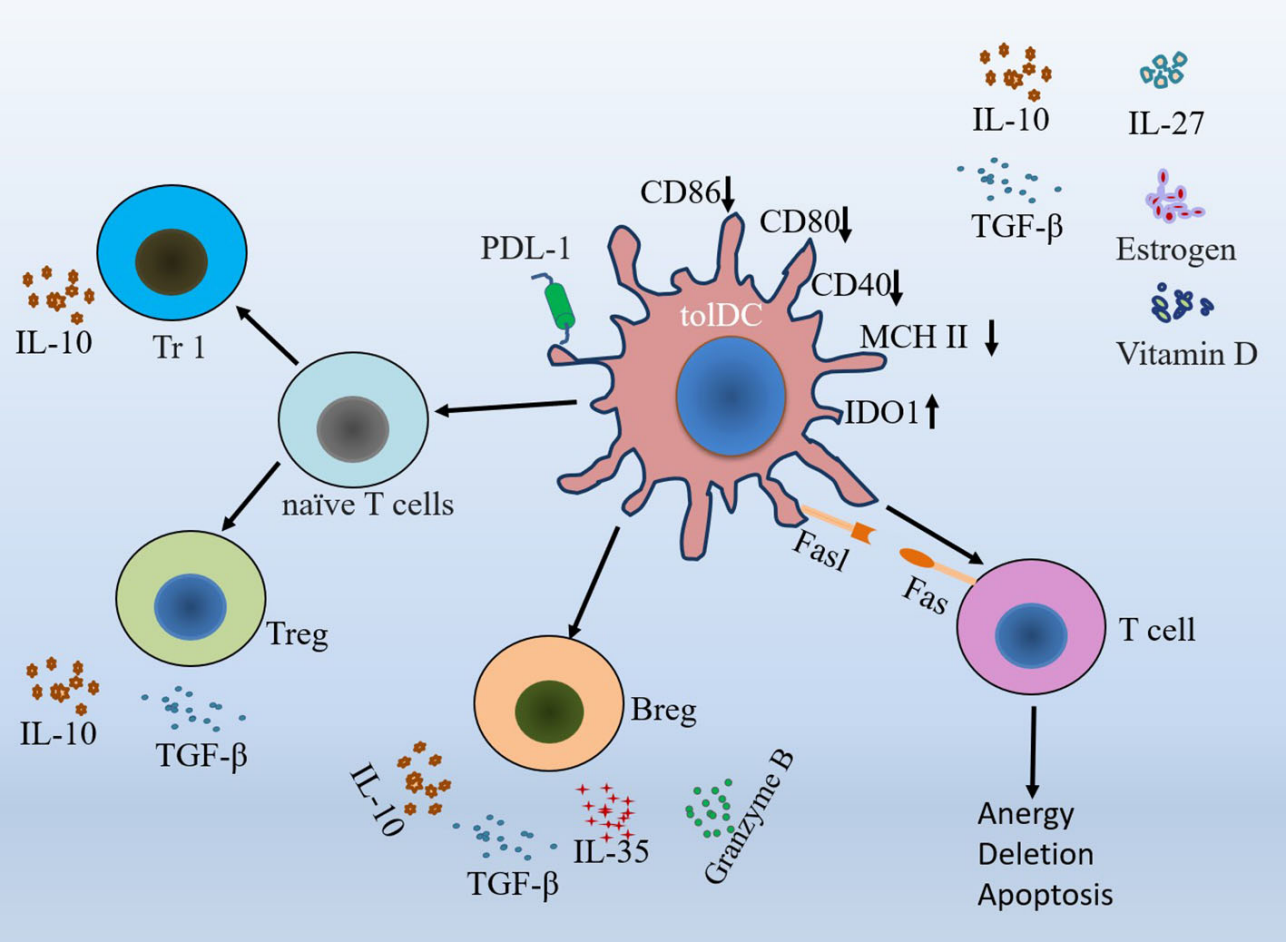
Mechanism of action of tolDCs in promoting tolerance.

TolDCs modulate immune tolerance by regulating the release of TGF-β, IL-10, IL-35, and granzyme B from regulatory B cells (Bregs) (44). IL-10 can also be secreted by Tregs and Tr1 cells, which are produced by tolDCs-induced naïve CD4 T cells (33, 48). Along with increasing IDO1 expression, tolDCs induced T cell apoptosis via the Fas/FasL pathway (27, 38). In addition, tolDCs release other cytokines to modulate T cell and Treg activity, and increase the expression and release of immunomodulatory molecules to promote the development of tolerance (38, 45). Perforin-expressing DCs, an important population of tolDCs, can restrict autoreactive T cells (2). Retinoic acid, directly secreted by tolDCs, inhibits effector T cells and induces Tregs and Bregs differentiation (49). CD103^+^ DCs, which produce TGFβ and retinoic acid, can induce the differentiation of naïve T cells into Tregs and Tr1 cells, thus further regulating the function of tolDCs (50). Heme oxygenase-1 (HO-1) regulates tolDCs function by actively inhibiting T cell responses. High expression of HO-1 favors the tolerance capacity of tolDCs, while when HO-1 is blocked, tolDCs lose their immunomodulatory effect (51). Vitamin D, estrogen, IL-27 and many others can also induce tolDCs to regulate immune tolerance (**Figure 2**) (33). Because they express low levels of costimulatory molecules and high levels of inhibitory receptors, tolDCs are beneficial in reducing inflammation and immune responses (18, 24).

## 4 TLR4/IRAK4/NF-κB signaling pathway

**Figure 3:**
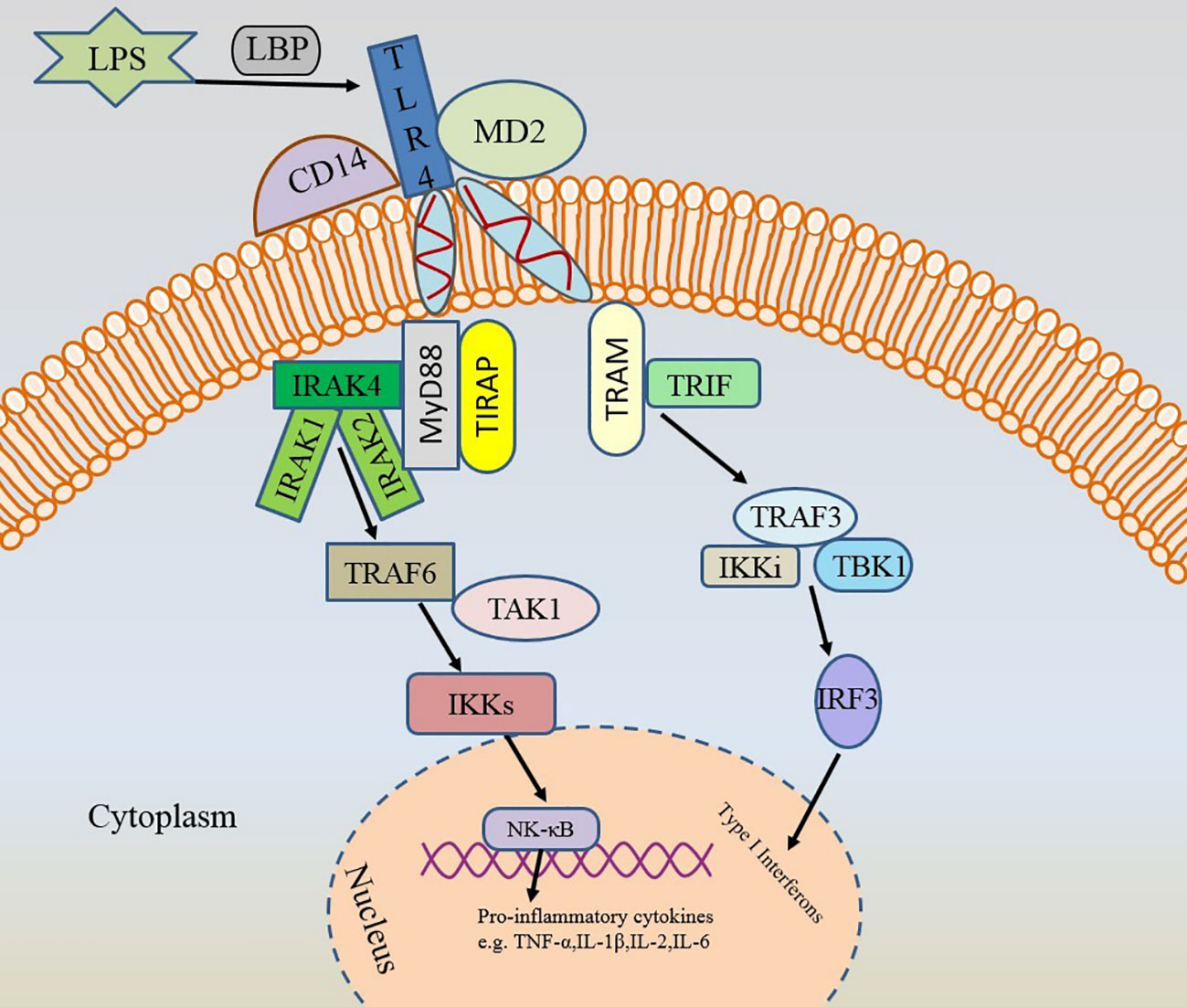
Two activation pathways of TLR4. TLR4, toll-like receptor 4; LPS, lipopolysaccharide; LBP, LPS-binding protein; MD-2, myeloid differentiation protein 2; MyD88, myeloid differentiation primary response 88; TIR, toll-interleukin-1 receptor; TRIF, TIR-domain-containing adapter-inducing interferon (IFN)-β; TIRAP, TIR domain-containing adaptor protein; TRAM, TRIF-related adaptor molecule; IRAK4, interleukin-1 receptor-associated kinase 4; TRAF6, TNF-receptor associated factor 6; IKK, IκB kinase; TAK1, transforming growth factor-activated kinase 1; NF-κB, nuclear factor kappa-B; IRF-3, IFN regulatory factor 3.

TLRs are 1 class of transmembrane proteins synthesized in the endoplasmic reticulum (52, 53). TLRs, sensing damage-associated molecular patterns (DAMPs) as well as PAMPs, can initiate signaling in both innate and adaptive immune pathways (54, 55). Up to now, 10 TLRs have been identified in humans and 12 in mice (56). Hyperactivation of TLR family member TLR4 triggers the production of inflammatory factors, which is associated with a variety of diseases (57). TLR4 triggers inflammation in various microbial infections, cancer, and autoimmune diseases (58). TLR4 induces pro-inflammatory responses to invading pathogens and plays a crucial role in allergic inflammation (59). In the presence of LPS-binding protein (LBP) and CD14, TLR4 binds to LPS with the help of co-receptor myeloid differentiation protein 2 (MD-2) (53, 59). The surroundings have an effect on the bond between them (60). Stimulated activation of TLR4 consists of two major intracellular signaling pathways: the myeloid differentiation primary response 88 (MyD88)-dependent pathway and the toll-interleukin-1 receptor (TIR) structural domain-containing adapter-induced IFN-β (TRIF) pathway (54). These two signaling pathways lead to the production of two sets of pro-inflammatory cytokines. Interaction of MyD88 and TIR homology domain-containing adaptor protein (TIRAP) further leads to activation of IRAK4, accompanied by activation of IRAK1 and IRAK2 (59, 61–63). IRAK4 is the most upstream kinase in this pathway and is directly related to MyD88 (61). IRAK4 plays a decisive role in the TIR signaling pathway (64). Activated IRAK4 is recruited to TNF-receptor associated factor 6 (TRAF6), which then further activates IκB kinase (IKK) signaling via transforming growth factor-activated kinase 1 (TAK1), ultimately leading to NF-κB activation and expression of other pro-inflammatory cytokines (59). These inflammatory factors will drive the inflammatory response, leading to hyperactivation of the immune system. While the TRIF pathway is mediated by TRIF and TRIF-related adaptor molecule (TRAM) to activate type 1 IFN genes and delayed NF-κB via IFN regulatory factor 3 (IRF-3) (54). These pathways will have an impact on the balance of inflammatory cytokines (**Figure 3**). Notably, TLR4 is the only TLR that relies on both MyD88 and TRIF pathways, and the TLR4/MyD88 signaling pathway is associated with AR (65, 66).

## 5 TolDCs and TLR4/IRAK4/NF-κB signaling pathway

DCs are one of the most important immune cells that express TLR4 (67). There are a large number of TLR4 receptors on the surface of DCs (68). Activation of the TLR4 signaling pathway promotes the maturation of DCs (67). The expression of costimulatory molecules CD80/CD86 on DCs is also affected by TLR4 (69). Meanwhile, TLR4 stimulates B cells to produce IL-10, which can further induce tolDCs (70). TLR4 expressed on DCs regulates immune tolerance by releasing IDO1, which further regulates adaptive immune responses by promoting immune suppression and tolerance (27, 67, 71). TLR4 ligation in LPS-primed DCs induced higher levels of IDO1 and aryl hydrocarbon receptor (AhR), further inducing the production of tolDCs (71). Cvetkovic et al. (72) showed that excretory-secretion products (ES L1) released by trichinella spiralis larvae induce the production of tolDCs via TLR4. Kim et al. (73) demonstrated that mycobacterium avium subspecies hominissuis (MAH) infection promotes the generation of tolDCs under the influence of TLR4 signaling. Han et al. (74) showed that minocycline may induce tolDCs by blocking the suppressor of cytokine signaling 1 (SOCS1)/TLR4/NF-κB signaling pathway. The above studies suggest that TLR4 may be a target for the action of tolDCs.

LPS primarily signals through TLR4 and thus may be involved in MyD88, IRAK4 and NF-κB signaling. As a cell wall component of all Gram-negative bacteria, LPS largely contributes to the induction of tolDCs by upregulating anti-inflammatory cytokines such as IL-10 and TGF-β (53, 75, 76). IL-10, which induces the generation of tolDCs, can inhibit IRAK4, TRAF6 and IRAK1 on the TLR4/IRAK4/NF-κB signaling pathway, thereby inhibiting MyD88-dependent TLR4 signaling (75). IL-10 also inhibits IKK, NF-κB P65/P50 activity (77). With the function of inducing tolDCs, IL-37 can combine with IL-18Rα to reduce the expression of MyD88, IRAK4 and TRAF6, which further leads to the reduction of NF-κB expression (78). MiRNA-155, miRNA-146, let-7 can regulate tolDCs via different signaling molecules, among which miR-146 can directly target IRAK1, TRAF6 to negatively regulate the TLR/MyD88/NF-κB pathway, and mi-155 can directly target TRAF6 (79, 80). Apoptotic cells induced the production of tolDCs to inhibit the transduction of the TLR4/NF-κB signaling pathway (81). Atorvastatin-induced tolDCs reduce inflammatory cell infiltration and inhibit oxidative stress via the TLR4/NF-κB signaling pathway (82).

NF-κB can be activated through canonical and non-canonical signaling pathways (83). Activation of the non-canonical NF-κB signaling pathway may be more effective in stimulating peripheral tolerance than the canonical NF-κB signaling pathway, which primarily responds to pro-inflammatory signals, and the non-canonical NF-κB pathway in tolDCs may treat inflammatory diseases (84). Inhibition of core transcription factor pathways such as NF-κB produces tolDCs, which further interfere with NF-κB signaling by increasing IL-10 (76, 85). The tolDCs phenotype is promoted by NF-κB p50, which negatively affects the survival of DCs and their ability to effectively activate T cells (75). The expression of tolerance-promoting molecules such as IDO1 can be enhanced and the expression of pro-inflammatory cytokines such as IFNβ, IL-1β, and IL-18 can be reduced during the accumulation of p50 in the nuclei of tolDCs (27, 75). The above studies demonstrated that tolDCs could inhibit different targets on the TLR4/IRAK4/NF-κB signaling pathway according to different induction signals. This will facilitate the development of different targeted drugs.

## 6 TolDCs and AR

DCs trigger allergic inflammation or contribute to immune tolerance to sensitizing allergens at different maturation stages, locations, and environments (86). The nature and level of inhaled allergens, route of administration, and changes in the local microenvironment all have an impact on the function of different subsets of DCs (5). Factors such as the type of antigen, the presence of danger signals in the microenvironment, and the genetic background of the host determine whether DCs produce strong Th2-driven allergic responses or acquire tolerance (5). TolDCs achieve suppressive function by suppressing T cell inflammation or activating Tregs (26). Induction of tolDCs by delivery of antibodies bearing the antigen has been shown to be highly efficient in ameliorating the disease process in a range of mouse models (4). Co-delivery of tolerogenic drugs and antigens into nanoparticles has been reported to promote the production of tolDCs (87).

TolDCs are beneficial in suppressing allergic inflammation and relieving allergic symptoms in AR, and may be a potential therapeutic target for AR. Suppression of allergic immune responses by tolDCs inducing allergen-specific blocking antibodies, immunosuppressive cytokines, Tregs, and Bregs is an alternative way to treat AR (88). Induction of tolDCs producing IDO1 promotes immune tolerance in allergic inflammation (27, 29). Upregulation of tolDCs and Tregs favors allergen-specific IgG production and induces immune tolerance, which may inhibit IgE activity and basophil activation (86). The protective allergen-specific IgG4 produced by Tregs competes with IgE for allergen binding to prevent IgE-mediated allergic reactions (52). TolDCs with high expression of IL-10 inhibit Th1 differentiation and limit effector T cell function, thereby suppressing allergic inflammation and promoting allergen-specific tolerance (41). Cui et al. (89) showed that activated AhR could induce the production of tolDCs to differentiate naïve T cells into Treg cells and inhibit Th17 cell differentiation, regulating the balance between Treg and Th17 cells. Activated AhR also induces tolDCs to generate Tr1 cells to regulate the balance of Th1 and Th17 (2). Min et al. (90) used LPS to activate bone marrow-derived DCs to induce the production of tolDCs in a mouse model *in vivo*, and they found that induced such tolDCs increased the number of Tregs in the lungs of ovalbumin-induced asthmatic mice, which could help to attenuate the Th2-mediated allergic immune response and treat allergic asthma. It is worth noting that the dosages of LPS need to be strictly controlled when inducing tolDCs, because the maturation of DCs by LPS is highly dose-dependent and also depends on the type of LPS used. Higher dosages induce an inflammatory DC phenotype rather than a tolerogenic one. Hong et al. (91) modulated immune tolerance by inducing tolDCs, which promotes the differentiation of Tregs thereby alleviating allergic reactions to food in mice. Sun et al. (46) demonstrated that Tregs are involved in the anti-inflammatory activity of tolDCs and that the adaptive transfer of tolDCs suppresses allergic airway inflammation. Liu et al. (92) showed that the protein disulfide isomerase (PDI) produced by house dust mites (HDMs) induces tolDCs to produce Tregs to promote immune tolerance, which helps to alleviate airway allergic inflammation. Liu et al. (93) showed that tolDCs and Tregs were suppressed in the AR nasal mucosa compared with those in the non-AR nasal mucosa, which in turn suggested that large amounts of tolDCs are beneficial in the treatment of AR. Sublingual immunotherapy (SLIT) of AR can also induce the production of tolDCs to further alleviate allergic inflammation (94). All these findings fully demonstrate the definitive efficacy of tolDCs in the treatment of allergic diseases. This will promote the application of tolDCs in the treatment of AR.

## 7 TLR4/IRAK4/NF-κB signaling pathway and AR

With abundant leucine repeats, TLRs regulate Th1/Th2 immune balance through DCs, mast cells and Tregs (52, 60). The adaptive immune response can be skewed toward Th1 by TLRs, which can cause DCs maturation and T cell activation (60). A variety of inflammatory cytokines and chemokines are released when TLRs activate DCs (95). Activation of DCs by allergens results in Th1/Th2 imbalance, which contributes to the development of AR. Genetic factors, environmental factors, and allergens themselves all influence the role of the TLRs in AR (52). TLRs agonists with well-defined immunomodulatory properties, favoring anti-allergic T lymphocyte responses and also increasing IL-10 production to prevent Th1 and Th17 responses (37, 96). TLR4 can initiate, exacerbate, or prevent allergic diseases (66). Generally, TLR4 is expressed at low levels and is upregulated once activated by allergens or other factors (52). TLR4 protein expression levels are elevated in the nasal mucosa of individuals with AR (96). Their elevated expression contributes to TLR4/MyD88 signaling to enhance inflammatory cell generation (66). MyD88 is a protein that articulates with TLR4 in the cytoplasm, which further mediates downstream signaling pathways.

IRAK4, a serine/threonine kinase, is an intermediate in the TLR4/NF-κB signaling pathway that transduces signals from TLR4 by bridging MyD88 (62, 97). IRAK4 is the only kinase in the IRAK family whose activity has been shown to be required to initiate signaling, and IRAK4 inhibitors will block all MyD88-dependent signaling (63). IRAK4 kinase has an important role in defense against infection *in vivo*, and its activity is a prerequisite for the establishment of an innate immune response, the loss of which will lead to exacerbation of the infection (98). Korppi et al. (99) showed that IRAK4 may play a role in allergic diseases, where IRAK4 rs4251513, rs4251559, and rs1461567 single nucleotide polymorphisms (SNPs) were associated with serum immunoglobulin E (IgE) levels. Staschke et al. (100) showed that IRAK4 kinase can regulate Th17 differentiation, thereby favoring the treatment of Th17-mediated inflammatory diseases, and their findings suggest that IRAK4 is a promising target for the treatment of Th17 cell-mediated inflammatory diseases. While Th17 cells are strongly associated with AR (101). Because of the important role of IRAK4 in triggering allergic inflammation, IRAK4 inhibitors could be targets for anti-inflammatory drugs (102, 103). Deletion or inactivation of IRAK4 attenuates the development of inflammation (104). Activation of IRAK4 transmits inflammatory signals to NF-κB in the nucleus via TRAF6 and IKKs.

NF-κB was originally thought to be a transcription factor that regulates immunoglobulin gene expression, and its optimal function may be to regulate the development and activation of the immune system (105). Further research has revealed that NF-κB is a key regulator of innate and adaptive immune responses (106). NF-κB is involved in signaling regulation of multiple pathways. Activation of NF-κB induces the secretion of many pro-inflammatory mediators leading to an inflammatory response as well as the activation of immune cells (107). DCs can recognize allergic inflammation and propagate pro-inflammatory signals through NF-κB, which can also influence the occurrence and development of allergic inflammation by regulating the differentiation and maturation of T cells (106). Increased NF-κB activity induces IgE synthesis, and decreased NF-κB activity suppresses allergic inflammatory responses (108, 109).

Multiple drugs can treat AR via TLR4/IRAK4/NF-κB signaling pathway. Wu et al. (110) showed that probiotics can ameliorate allergic inflammation through the TLR4/NF-κB signaling pathway. Dong et al. (111) showed that Luteolin could treat AR by improving Th1/Th2 imbalance and reducing inflammation via the TLR4/NF-κB signaling pathway. Li et al. (112) showed that apigenin could attenuate the inflammatory response in AR through the TLR4/MyD88/NF-κB signaling pathway. Liu et al. (113) showed that microRNA-345-5p could alleviate allergic inflammation in AR mice through the TLR4/NF-κB signaling pathway. These studies amply demonstrate the inseparable relationship between the TLR4/NF-κB signaling pathway and AR. We can choose the best target on this signaling pathway to intervene in AR depending on the needs.

## 8 Conclusions and future outlook

The immune response of DCs in AR towards immunity or tolerance is essential for the treatment of AR. We can induce DCs with tolerance function on demand, and inducing tolDCs to act on specific targets is expected to be a scalable immunotherapy for AR. Knocking down the expression of costimulatory molecules and MHC class II with inhibitors of the TLR4/IRAK4/NF-κB signaling pathway to induce tolDCs is a feasible approach. NF-κB inhibitors that induce tolDCs are already in clinical trials (45). We can also combine the immunogenicity and tolerability of DCs as needed for better clinical translation. The purpose of inducing tolDCs is to suppress unwanted immune responses in the long term (24). DC-targeting strategies reduce the risk of extensive immunosuppression (4). Current studies have shown that the application of DCs immunotherapy is safe and well tolerated (114).

Despite the great potential of tolDCs-based immunotherapies, the mechanism of their immunomodulatory activity is unclear (18), and further studies and a full understanding of their function in immunosuppression are needed. Are tolDCs phenotypically and functionally stable in a pro-inflammatory environment? Whether tolDCs have a stable and long-lasting effect in treating AR via the TLR4/IRAK4/NF-κB signaling pathway? This is something we need to explore further. Although there are many ways for inducing tolDCs, there are still substantial knowledge gaps to be filled in the application of tolDCs to treat AR via the TLR4/IRAK4NF-κB signaling pathway. Easier, shorter cycles and long-term tolerance are our goals. A better understanding of the pathogenesis of AR will contribute to new ways of treating AR. We hope to use tolDCs to develop drugs without toxic side effects to treat AR via the TLR4/IRAK4NF-κB signaling pathway in the future.

In conclusion, the potential of tolDCs applied to the treatment of AR is enormous.

## Author contributions

CK: Writing – original draft. XL: Writing – original draft. PL: Writing – review & editing. YL: Writing – review & editing. YN: Writing – review & editing. XZ: Writing – review & editing. HZ: Supervision, Writing – review & editing. JL: Funding acquisition, Supervision, Writing – review & editing. SQ: Supervision, Writing – review & editing.

## References

[1] Balan S Saxena M Bhardwaj N. Dendritic cell subsets and locations. Int Rev Cell Mol Biol (2019) 348:1–68. doi: 10.1016/bs.ircmb.2019.07.004 31810551

[2] Takenaka MC Quintana FJ. Tolerogenic dendritic cells. Semin Immunopathol (2017) 39(2):113–20. doi: 10.1007/s00281-016-0587-8 PMC5296314 27646959

[3] Waisman A Lukas D Clausen BE Yogev N. Dendritic cells as gatekeepers of tolerance. Semin Immunopathol (2017) 39(2):153–63. doi: 10.1007/s00281-016-0583-z 27456849

[4] Castenmiller C Keumatio-Doungtsop BC van Ree R de Jong EC van Kooyk Y. Tolerogenic immunotherapy: targeting DC surface receptors to induce antigen-specific tolerance. Front Immunol (2021) 12:643240. doi: 10.3389/fimmu.2021.643240 33679806 PMC7933040

[5] Morianos I Semitekolou M. Dendritic cells: critical regulators of allergic asthma. Int J Mol Sci (2020) 21(21):7930. doi: 10.3390/ijms21217930 33114551 PMC7663753

[6] Bousquet J Anto JM Bachert C Baiardini I Bosnic-Anticevich S Walter Canonica G. Allergic rhinitis. Nat Rev Dis Primers. (2020) 6(1):95. doi: 10.1038/s41572-020-00227-0 33273461

[7] Luckey U Maurer M Schmidt T Lorenz N Seebach B Metz M. T cell killing by tolerogenic dendritic cells protects mice from allergy. J Clin Invest. (2011) 121(10):3860–71. doi: 10.1172/JCI45963 PMC3195460 21881208

[8] Hollingsworth JW Free ME Li Z Andrews LN Nakano H Cook DN. Ozone activates pulmonary dendritic cells and promotes allergic sensitization through a Toll-like receptor 4-dependent mechanism. J Allergy Clin Immunol (2010) 125(5):1167–70. doi: 10.1016/j.jaci.2010.03.001 PMC2866779 20394980

[9] Feng M Zhou S Yu Y Su Q Li X Lin W. Regulation of the migration of distinct dendritic cell subsets. Front Cell Dev Biol (2021) 9:635221. doi: 10.3389/fcell.2021.635221 33681216 PMC7933215

[10] Kuzmich NN Sivak KV Chubarev VN Porozov YB Savateeva-Lyubimova TN Peri F. TLR4 signaling pathway modulators as potential therapeutics in inflammation and sepsis. Vaccines (2017) 5(4):34. doi: 10.3390/vaccines5040034 28976923 PMC5748601

[11] Eisenbarth SC. Dendritic cell subsets in T cell programming: location dictates function. Nat Rev Immunol (2019) 19(2):89–103. doi: 10.1038/s41577-018-0088-1 30464294 PMC7755085

[12] Reizis B. Plasmacytoid dendritic cells: development, regulation, and function. Immunity (2019) 50(1):37–50. doi: 10.1016/j.immuni.2018.12.027 30650380 PMC6342491

[13] Swiecki M Colonna M. The multifaceted biology of plasmacytoid dendritic cells. Nat Rev Immunol (2015) 15(8):471–85. doi: 10.1038/nri3865 PMC4808588 26160613

[14] Murphy TL Murphy KM. Dendritic cells in cancer immunology. Cell Mol Immunol (2022) 19(1):3–13. doi: 10.1038/s41423-021-00741-5 34480145 PMC8752832

[15] Hilligan KL Ronchese F. Antigen presentation by dendritic cells and their instruction of CD4+ T helper cell responses. Cell Mol Immunol (2020) 17(6):587–99. doi: 10.1038/s41423-020-0465-0 PMC7264306 32433540

[16] Gargaro M Scalisi G Manni G Briseño CG Bagadia P Durai V. Indoleamine 2,3-dioxygenase 1 activation in mature cDC1 promotes tolerogenic education of inflammatory cDC2 *via* metabolic communication. Immunity (2022) 55(6):1032–50.e14. doi: 10.1016/j.immuni.2022.05.013 35704993 PMC9220322

[17] Binnewies M Mujal AM Pollack JL Combes AJ Hardison EA Barry KC. Unleashing type-2 dendritic cells to drive protective antitumor CD4(+) T cell immunity. Cell (2019) 177(3):556–71.e16. doi: 10.1016/j.cell.2019.02.005 30955881 PMC6954108

[18] Nam JH Lee JH Choi SY Jung NC Song JY Seo HG. Functional ambivalence of dendritic cells: tolerogenicity and immunogenicity. Int J Mol Sci (2021) 22(9):4430. doi: 10.3390/ijms22094430 33922658 PMC8122871

[19] Sabado RL Bhardwaj N. Dendritic cell immunotherapy. Ann N Y Acad Sci (2013) 1284:31–45. doi: 10.1111/nyas.12125 23651191

[20] Yu H Tian Y Wang Y Mineishi S Zhang Y. Dendritic cell regulation of graft-vs.-host disease: immunostimulation and tolerance. Front Immunol (2019) 10:93. doi: 10.3389/fimmu.2019.00093 30774630 PMC6367268

[21] Tiberio L Del Prete A Schioppa T Sozio F Bosisio D Sozzani S. Chemokine and chemotactic signals in dendritic cell migration. Cell Mol Immunol (2018) 15(4):346–52. doi: 10.1038/s41423-018-0005-3 PMC6052805 29563613

[22] Worbs T Hammerschmidt SI Förster R. Dendritic cell migration in health and disease. Nat Rev Immunol (2017) 17(1):30–48. doi: 10.1038/nri.2016.116 27890914

[23] Maldonado RA von Andrian UH. How tolerogenic dendritic cells induce regulatory T cells. Adv Immunol (2010) 108:111–65. doi: 10.1016/B978-0-12-380995-7.00004-5 PMC3050492 21056730

[24] Passeri L Marta F Bassi V Gregori S. Tolerogenic dendritic cell-based approaches in autoimmunity. Int J Mol Sci (2021) 22(16):8415. doi: 10.3390/ijms22168415 34445143 PMC8395087

[25] Hadeiba H Butcher EC. Thymus-homing dendritic cells in central tolerance. Eur J Immunol (2013) 43(6):1425–9. doi: 10.1002/eji.201243192 PMC3774955 23616226

[26] Devi KS Anandasabapathy N. The origin of DCs and capacity for immunologic tolerance in central and peripheral tissues. Semin Immunopathol (2017) 39(2):137–52. doi: 10.1007/s00281-016-0602-0 PMC5296242 27888331

[27] Merlo LMF Peng W Mandik-Nayak L. Impact of IDO1 and IDO2 on the B cell immune response. Front Immunol (2022) 13:886225. doi: 10.3389/fimmu.2022.886225 35493480 PMC9043893

[28] Ganguly D Haak S Sisirak V Reizis B. The role of dendritic cells in autoimmunity. Nat Rev Immunol (2013) 13(8):566–77. doi: 10.1038/nri3477 PMC4160805 23827956

[29] Horwitz DA Fahmy TM Piccirillo CA La Cava A. Rebalancing immune homeostasis to treat autoimmune diseases. Trends Immunol (2019) 40(10):888–908. doi: 10.1016/j.it.2019.08.003 31601519 PMC7136015

[30] Audiger C Rahman MJ Yun TJ Tarbell KV Lesage S. The importance of dendritic cells in maintaining immune tolerance. J Immunol (2017) 198(6):2223–31. doi: 10.4049/jimmunol.1601629 PMC5343761 28264998

[31] Li S Wu J Zhu S Liu YJ Chen J. Disease-associated plasmacytoid dendritic cells. Front Immunol (2017) 8:1268. doi: 10.3389/fimmu.2017.01268 29085361 PMC5649186

[32] Shortman K Liu YJ. Mouse and human dendritic cell subtypes. Nat Rev Immunol (2002) 2(3):151–61. doi: 10.1038/nri746 11913066

[33] Xie ZX Zhang HL Wu XJ Zhu J Ma DH Jin T. Role of the immunogenic and tolerogenic subsets of dendritic cells in multiple sclerosis. Mediators Inflamm (2015) 2015:513295. doi: 10.1155/2015/513295 25705093 PMC4325219

[34] Sim WJ Ahl PJ Connolly JE. Metabolism is central to tolerogenic dendritic cell function. Mediators Inflamm (2016) 2016:2636701. doi: 10.1155/2016/2636701 26980944 PMC4766347

[35] Deak P Knight HR Esser-Kahn A. Robust tolerogenic dendritic cells *via* push/pull pairing of toll-like-receptor agonists and immunomodulators reduces EAE. Biomaterials (2022) 286:121571. doi: 10.1016/j.biomaterials.2022.121571 35597168 PMC10152544

[36] Sahar A Nicorescu I Barran G Paterson M Hilkens CMU Lord P. Tolerogenic dendritic cell reporting: Has a minimum information model made a difference? PeerJ (2023) 11:e15352. doi: 10.7717/peerj.15352 37273539 PMC10239229

[37] Iberg CA Hawiger D. Natural and induced tolerogenic dendritic cells. J Immunol (2020) 204(4):733–44. doi: 10.4049/jimmunol.1901121 PMC7006631 32015076

[38] Ochando J Ordikhani F Jordan S Boros P Thomson AW. Tolerogenic dendritic cells in organ transplantation. Transpl Int (2020) 33(2):113–27. doi: 10.1111/tri.13504 PMC6983332 31472079

[39] Funes SC Manrique de Lara A Altamirano-Lagos MJ Mackern-Oberti JP Escobar-Vera J Kalergis AM. Immune checkpoints and the regulation of tolerogenicity in dendritic cells: Implications for autoimmunity and immunotherapy. Autoimmun Rev (2019) 18(4):359–68. doi: 10.1016/j.autrev.2019.02.006 30738957

[40] Hu J Wan Y. Tolerogenic dendritic cells and their potential applications. Immunology (2011) 132(3):307–14. doi: 10.1111/j.1365-2567.2010.03396.x PMC3044897 21208205

[41] Schülke S. Induction of interleukin-10 producing dendritic cells as a tool to suppress allergen-specific T helper 2 responses. Front Immunol (2018) 9:455. doi: 10.3389/fimmu.2018.00455 29616018 PMC5867300

[42] Fucikova J Palova-Jelinkova L Bartunkova J Spisek R. Induction of tolerance and immunity by dendritic cells: mechanisms and clinical applications. Front Immunol (2019) 10:2393. doi: 10.3389/fimmu.2019.02393 31736936 PMC6830192

[43] Cifuentes-Rius A Desai A Yuen D Johnston APR Voelcker NH. Inducing immune tolerance with dendritic cell-targeting nanomedicines. Nat Nanotechnol. (2021) 16(1):37–46. doi: 10.1038/s41565-020-00810-2 33349685

[44] Marín E Cuturi MC Moreau A. Tolerogenic dendritic cells in solid organ transplantation: where do we stand? Front Immunol (2018) 9:274. doi: 10.3389/fimmu.2018.00274 29520275 PMC5827529

[45] Zhuang Q Cai H Cao Q Li Z Liu S Ming Y. Tolerogenic dendritic cells: the pearl of immunotherapy in organ transplantation. Front Immunol (2020) 11:552988. doi: 10.3389/fimmu.2020.552988 33123131 PMC7573100

[46] Sun W Wei JW Li H Wei FQ Li J Wen WP. Adoptive cell therapy of tolerogenic dendritic cells as inducer of regulatory T cells in allergic rhinitis. Int Forum Allergy Rhinol (2018) 8(11):1291–9. doi: 10.1002/alr.22217 30281915

[47] Durham SR Shamji MH. Allergen immunotherapy: past, present and future. Nat Rev Immunol (2023) 23(5):317–28. doi: 10.1038/s41577-022-00786-1 PMC9575636 36253555

[48] Raffin C Vo LT Bluestone JA. T(reg) cell-based therapies: challenges and perspectives. Nat Rev Immunol (2020) 20(3):158–72. doi: 10.1038/s41577-019-0232-6 PMC7814338 31811270

[49] Morante-Palacios O Fondelli F Ballestar E Martínez-Cáceres EM. Tolerogenic dendritic cells in autoimmunity and inflammatory diseases. Trends Immunol (2021) 42(1):59–75. doi: 10.1016/j.it.2020.11.001 33293219

[50] Yu W Freeland DMH Nadeau KC. Food allergy: immune mechanisms, diagnosis and immunotherapy. Nat Rev Immunol (2016) 16(12):751–65. doi: 10.1038/nri.2016.111 PMC5123910 27795547

[51] Zhao Y Jia Y Wang L Chen S Huang X Xu B. Upregulation of heme oxygenase-1 endues immature dendritic cells with more potent and durable immunoregulatory properties and promotes engraftment in a stringent mouse cardiac allotransplant model. Front Immunol (2018) 9:1515. doi: 10.3389/fimmu.2018.01515 30013566 PMC6036127

[52] Wenger M Grosse-Kathoefer S Kraiem A Pelamatti E Nunes N Pointner L. When the allergy alarm bells toll: The role of Toll-like receptors in allergic diseases and treatment. Front Mol Biosci (2023) 10:1204025. doi: 10.3389/fmolb.2023.1204025 37426425 PMC10325731

[53] Fitzgerald KA Kagan JC. Toll-like receptors and the control of immunity. Cell (2020) 180(6):1044–66. doi: 10.1016/j.cell.2020.02.041 PMC9358771 32164908

[54] Bruno K Woller SA Miller YI Yaksh TL Wallace M Beaton G. Targeting toll-like receptor-4 (TLR4)-an emerging therapeutic target for persistent pain states. Pain (2018) 159(10):1908–15. doi: 10.1097/j.pain.0000000000001306 PMC7890571 29889119

[55] Arora S Ahmad S Irshad R Goyal Y Rafat S Siddiqui N. TLRs in pulmonary diseases. Life Sci (2019) 233:116671. doi: 10.1016/j.lfs.2019.116671 31336122 PMC7094289

[56] Duan T Du Y Xing C Wang HY Wang RF. Toll-like receptor signaling and its role in cell-mediated immunity. Front Immunol (2022) 13:812774. doi: 10.3389/fimmu.2022.812774 35309296 PMC8927970

[57] Zhang Y Liang X Bao X Xiao W Chen G. Toll-like receptor 4 (TLR4) inhibitors: Current research and prospective. Eur J Med Chem (2022) 235:114291. doi: 10.1016/j.ejmech.2022.114291 35307617

[58] Mahish C De S Chatterjee S Ghosh S Keshry SS Mukherjee T. TLR4 is one of the receptors for Chikungunya virus envelope protein E2 and regulates virus induced pro-inflammatory responses in host macrophages. Front Immunol (2023) 14:1139808. doi: 10.3389/fimmu.2023.1139808 37153546 PMC10157217

[59] Ciesielska A Matyjek M Kwiatkowska K. TLR4 and CD14 trafficking and its influence on LPS-induced pro-inflammatory signaling. Cell Mol Life Sci (2021) 78(4):1233–61. doi: 10.1007/s00018-020-03656-y PMC7904555 33057840

[60] Chen RX Dai MD Zhang QZ Lu MP Wang ML Yin M. TLR signaling pathway gene polymorphisms, gene-gene and gene-environment interactions in allergic rhinitis. J Inflammation Res (2022) 15:3613–30. doi: 10.2147/JIR.S364877 PMC9234183 35769128

[61] Wang L Ferrao R Li Q Hatcher JM Choi HG Buhrlage SJ. Conformational flexibility and inhibitor binding to unphosphorylated interleukin-1 receptor-associated kinase 4 (IRAK4). J Biol Chem (2019) 294(12):4511–9. doi: 10.1074/jbc.RA118.005428 PMC6433055 30679311

[62] Pereira M Durso DF Bryant CE Kurt-Jones EA Silverman N Golenbock DT. The IRAK4 scaffold integrates TLR4-driven TRIF and MYD88 signaling pathways. Cell Rep (2022) 40(7):111225. doi: 10.1016/j.celrep.2022.111225 35977521 PMC9446533

[63] Sabnis RW. Novel tricyclic heteroaryl compounds as IRAK4 inhibitors for treating cancer, autoimmune and inflammatory diseases. ACS Med Chem Lett (2022) 13(3):336–7. doi: 10.1021/acsmedchemlett.2c00060 PMC8919273 35300088

[64] Seumen CHT Grimm TM Hauck CR. Protein phosphatases in TLR signaling. Cell Commun Signal (2021) 19(1):45. doi: 10.1186/s12964-021-00722-1 33882943 PMC8058998

[65] Squillace S Salvemini D. Toll-like receptor-mediated neuroinflammation: relevance for cognitive dysfunctions. Trends Pharmacol Sci (2022) 43(9):726–39. doi: 10.1016/j.tips.2022.05.004 PMC9378500 35753845

[66] Shalaby KH Al Heialy S Tsuchiya K Farahnak S McGovern TK Risse PA. The TLR4-TRIF pathway can protect against the development of experimental allergic asthma. Immunology (2017) 152(1):138–49. doi: 10.1111/imm.12755 PMC5543728 28502093

[67] Bruning EE Coller JK Wardill HR Bowen JM. Site-specific contribution of Toll-like receptor 4 to intestinal homeostasis and inflammatory disease. J Cell Physiol (2021) 236(2):877–88. doi: 10.1002/jcp.29976 32730645

[68] Ye Z Zhong L Zhu S Wang Y Zheng J Wang S. The P-selectin and PSGL-1 axis accelerates atherosclerosis *via* activation of dendritic cells by the TLR4 signaling pathway. Cell Death Dis (2019) 10(7):507. doi: 10.1038/s41419-019-1736-5 31263109 PMC6602970

[69] Pufnock JS Cigal M Rolczynski LS Andersen-Nissen E Wolfl M McElrath MJ. Priming CD8+ T cells with dendritic cells matured using TLR4 and TLR7/8 ligands together enhances generation of CD8+ T cells retaining CD28. Blood (2011) 117(24):6542–51. doi: 10.1182/blood-2010-11-317966 PMC3123022 21493800

[70] Boldison J Da Rosa LC Davies J Wen L Wong FS. Dendritic cells license regulatory B cells to produce IL-10 and mediate suppression of antigen-specific CD8 T cells. Cell Mol Immunol (2020) 17(8):843–55. doi: 10.1038/s41423-019-0324-z PMC7395736 31728048

[71] Salazar F Awuah D Negm OH Shakib F Ghaemmaghami AM. The role of indoleamine 2,3-dioxygenase-aryl hydrocarbon receptor pathway in the TLR4-induced tolerogenic phenotype in human DCs. Sci Rep (2017) 7:43337. doi: 10.1038/srep43337 28256612 PMC5335671

[72] Cvetkovic J Ilic N Gruden-Movsesijan A Tomic S Mitic N Pinelli E. DC-SIGN signalling induced by Trichinella spiralis products contributes to the tolerogenic signatures of human dendritic cells. Sci Rep (2020) 10(1):20283. doi: 10.1038/s41598-020-77497-x 33219293 PMC7679451

[73] Kim WS Yoon JH Shin MK Shin SJ. Infection of Dendritic Cells With Mycobacterium avium subspecies hominissuis Exhibits a Functionally Tolerogenic Phenotype in Response to Toll-Like Receptor Agonists *via* IL-10/Cox2/PGE2/EP2 Axis. Front Microbiol (2019) 10:1795. doi: 10.3389/fmicb.2019.01795 31440223 PMC6692481

[74] Han X Wei Q Xu RX Wang S Liu XY Guo C. Minocycline induces tolerance to dendritic cell production probably by targeting the SOCS1/ TLR4/NF-κB signaling pathway. Transpl Immunol (2023) 79:101856. doi: 10.1016/j.trim.2023.101856 37196867

[75] Steimle A Frick JS. Molecular mechanisms of induction of tolerant and tolerogenic intestinal dendritic cells in mice. J Immunol Res (2016) 2016:1958650. doi: 10.1155/2016/1958650 26981546 PMC4766351

[76] Klaska IP Muckersie E Martin-Granados C Christofi M Forrester JV. Lipopolysaccharide-primed heterotolerant dendritic cells suppress experimental autoimmune uveoretinitis by multiple mechanisms. Immunology (2017) 150(3):364–77. doi: 10.1111/imm.12691 PMC5290303 27859049

[77] Hubo M Trinschek B Kryczanowsky F Tuettenberg A Steinbrink K Jonuleit H. Costimulatory molecules on immunogenic versus tolerogenic human dendritic cells. Front Immunol (2013) 4:82. doi: 10.3389/fimmu.2013.00082 23565116 PMC3615188

[78] Dinarello CA Nold-Petry C Nold M Fujita M Li S Kim S. Suppression of innate inflammation and immunity by interleukin-37. Eur J Immunol (2016) 46(5):1067–81. doi: 10.1002/eji.201545828 PMC5003108 27060871

[79] Zheng J Jiang HY Li J Tang HC Zhang XM Wang XR. MicroRNA-23b promotes tolerogenic properties of dendritic cells in *vitro* through inhibiting Notch1/NF-κB signalling pathways. Allergy (2012) 67(3):362–70. doi: 10.1111/j.1398-9995.2011.02776.x 22229716

[80] Kim SJ Diamond B. Modulation of tolerogenic dendritic cells and autoimmunity. Semin Cell Dev Biol (2015) 41:49–58. doi: 10.1016/j.semcdb.2014.04.020 24747368 PMC9973561

[81] Trahtemberg U Mevorach D. Apoptotic cells induced signaling for immune homeostasis in macrophages and dendritic cells. Front Immunol (2017) 8:1356. doi: 10.3389/fimmu.2017.01356 29118755 PMC5661053

[82] Wang Q Chen Z Guo J Peng X Zheng Z Chen H. Atorvastatin-induced tolerogenic dendritic cells improve cardiac remodeling by suppressing TLR-4/NF-κB activation after myocardial infarction. Inflammation Res (2023) 72(1):13–25. doi: 10.1007/s00011-022-01654-3 36315279

[83] Sun SC. The non-canonical NF-κB pathway in immunity and inflammation. Nat Rev Immunol (2017) 17(9):545–58. doi: 10.1038/nri.2017.52 PMC5753586 28580957

[84] van Delft MA Huitema LF Tas SW. The contribution of NF-κB signalling to immune regulation and tolerance. Eur J Clin Invest. (2015) 45(5):529–39. doi: 10.1111/eci.12430 25735405

[85] Jha A Ahad A Mishra GP Sen K Smita S Minz AP. SMRT and NCoR1 fine-tune inflammatory versus tolerogenic balance in dendritic cells by differentially regulating STAT3 signaling. Front Immunol (2022) 13:910705. doi: 10.3389/fimmu.2022.910705 36238311 PMC9552960

[86] Wang W Yin J. Is it worthy to take full-course immunotherapy for allergic rhinitis? About efficacy biomarker of allergen immunotherapy. Scand J Immunol (2020) 91(1):e12817. doi: 10.1111/sji.12817 31650620

[87] Stead SO Kireta S McInnes SJP Kette FD Sivanathan KN Kim J. Murine and non-human primate dendritic cell targeting nanoparticles for in vivo generation of regulatory T-cells. ACS nano. (2018) 12(7):6637–47. doi: 10.1021/acsnano.8b01625 29979572

[88] Drazdauskaitė G Layhadi JA Shamji MH. Mechanisms of allergen immunotherapy in allergic rhinitis. Curr Allergy Asthma Rep (2020) 21(1):2. doi: 10.1007/s11882-020-00977-7 33313967 PMC7733588

[89] Cui X Ye Z Wang D Yang Y Jiao C Ma J. Aryl hydrocarbon receptor activation ameliorates experimental colitis by modulating the tolerogenic dendritic and regulatory T cell formation. Cell Biosci (2022) 12(1):46. doi: 10.1186/s13578-022-00780-z 35461286 PMC9034494

[90] Min Z Zeng Y Zhu T Cui B Mao R Jin M. Lipopolysaccharide-activated bone marrow-derived dendritic cells suppress allergic airway inflammation by ameliorating the immune microenvironment. Front Immunol (2021) 12:595369. doi: 10.3389/fimmu.2021.595369 34093516 PMC8171252

[91] Hong J Xiao X Gao Q Li S Jiang B Sun X. Co-delivery of allergen epitope fragments and R848 inhibits food allergy by inducing tolerogenic dendritic cells and regulatory T cells. Int J Nanomedicine. (2019) 14:7053–64. doi: 10.2147/IJN.S215415 PMC6722440 31564865

[92] Liu X Wang Y Chen D Ji S Yang LT Huang Q. Dust-mite-derived protein disulfide isomerase suppresses airway allergy by inducing tolerogenic dendritic cells. J Biol Chem (2021) 296:100585. doi: 10.1016/j.jbc.2021.100585 33771560 PMC8080076

[93] Liu T Liang X Li TL Ma J Yang JF Yang PC. Staphylococcal enterotoxin B compromises the immune tolerant status in the airway mucosa. Clin Exp Allergy (2012) 42(3):375–82. doi: 10.1111/j.1365-2222.2011.03869.x 22093045

[94] Moingeon P. Update on immune mechanisms associated with sublingual immunotherapy: practical implications for the clinician. J Allergy Clin Immunol Pract (2013) 1(3):228–41. doi: 10.1016/j.jaip.2013.03.013 24565479

[95] Napolitani G Rinaldi A Bertoni F Sallusto F Lanzavecchia A. Selected Toll-like receptor agonist combinations synergistically trigger a T helper type 1-polarizing program in dendritic cells. Nat Immunol (2005) 6(8):769–76. doi: 10.1038/ni1223 PMC3760217 15995707

[96] Kirtland ME Tsitoura DC Durham SR Shamji MH. Toll-like receptor agonists as adjuvants for allergen immunotherapy. Front Immunol (2020) 11:599083. doi: 10.3389/fimmu.2020.599083 33281825 PMC7688745

[97] Li Q Li R Yin H Wang S Liu B Li J. Oral IRAK4 inhibitor BAY-1834845 prevents acute respiratory distress syndrome. BioMed Pharmacother. (2022) 153:113459. doi: 10.1016/j.biopha.2022.113459 36076574 PMC9339262

[98] Pattabiraman G Murphy M Agliano F Karlinsey K Medvedev AE. IRAK4 activity controls immune responses to intracellular bacteria Listeria monocytogenes and Mycobacterium smegmatis. J Leukoc Biol (2018) 104(4):811–20. doi: 10.1002/JLB.2A1117-449R PMC6162151 29749650

[99] Korppi M Teräsjärvi J Lauhkonen E Törmänen S He Q Nuolivirta K. Interleukin-1 receptor-associated kinase-4 gene variation may increase post-bronchiolitis asthma risk. Acta Paediatr (2021) 110(3):952–8. doi: 10.1111/apa.15607 33020954

[100] Staschke KA Dong S Saha J Zhao J Brooks NA Hepburn DL. IRAK4 kinase activity is required for Th17 differentiation and Th17-mediated disease. J Immunol (2009) 183(1):568–77. doi: 10.4049/jimmunol.0802361 PMC3638260 19542468

[101] Ueda S Miura K Kawasaki H Ogata S Yamasaki N Miura S. Th17-dependent nasal hyperresponsiveness is mitigated by steroid treatment. Biomolecules (2022) 12(5):674. doi: 10.3390/biom12050674 35625602 PMC9138412

[102] Sabnis RW. Novel IRAK4 inhibitors for treating asthma. ACS Med Chem Lett (2022) 13(8):1219–20. doi: 10.1021/acsmedchemlett.2c00324 PMC9377324 35978689

[103] Otto G. IRAK4 inhibitor attenuates inflammation. Nat Rev Rheumatol (2021) 17(11):646. doi: 10.1038/s41584-021-00699-8 34584262

[104] Winkler A Sun W De S Jiao A Sharif MN Symanowicz PT. The interleukin-1 receptor-associated kinase 4 inhibitor PF-06650833 blocks inflammation in preclinical models of rheumatic disease and in humans enrolled in a randomized clinical trial. Arthritis Rheumatol (2021) 73(12):2206–18. doi: 10.1002/art.41953 PMC8671219 34423919

[105] Sun SC Liu ZG. A special issue on NF-κB signaling and function. Cell Res (2011) 21(1):1–2. doi: 10.1038/cr.2011.1 21196938 PMC3193411

[106] Hayden MS Ghosh S. NF-κB in immunobiology. Cell Res (2011) 21(2):223–44. doi: 10.1038/cr.2011.13 PMC3193440 21243012

[107] Yu H Lin L Zhang Z Zhang H Hu H. Targeting NF-κB pathway for the therapy of diseases: mechanism and clinical study. Signal Transduct Target Ther (2020) 5(1):209. doi: 10.1038/s41392-020-00312-6 32958760 PMC7506548

[108] Yanagihara Y Basaki Y Ikizawa K Kajiwara K Koshio T Akiyama K. Involvement of nuclear factor-kappa B activation in IgE synthesis in human B cells. J Allergy Clin Immunol (1996) 98(6 Pt 2):S224–9. doi: 10.1016/S0091-6749(96)70070-2 8977531

[109] Piao CH Fan YJ Nguyen TV Song CH Chai OH. Mangiferin alleviates ovalbumin-induced allergic rhinitis *via* nrf2/HO-1/NF-κB signaling pathways. Int J Mol Sci (2020) 21(10):3415. doi: 10.3390/ijms21103415 32408566 PMC7279452

[110] Wu Z Mehrabi Nasab E Arora P Athari SS. Study effect of probiotics and prebiotics on treatment of OVA-LPS-induced of allergic asthma inflammation and pneumonia by regulating the TLR4/NF-kB signaling pathway. J Transl Med (2022) 20(1):130. doi: 10.1186/s12967-022-03337-3 35296330 PMC8925173

[111] Dong J Xu O Wang J Shan C Ren X. Luteolin ameliorates inflammation and Th1/Th2 imbalance *via* regulating the TLR4/NF-κB pathway in allergic rhinitis rats. Immunopharmacol Immunotoxicol. (2021) 43(3):319–27. doi: 10.1080/08923973.2021.1905659 33900898

[112] Li H Zhang H Zhao H. Apigenin attenuates inflammatory response in allergic rhinitis mice by inhibiting the TLR4/MyD88/NF-κB signaling pathway. Environ Toxicol (2023) 38(2):253–65. doi: 10.1002/tox.23699 36350155

[113] Liu J Jiang Y Han M Jiang L Liang D Li S. MicroRNA-345-5p acts as an anti-inflammatory regulator in experimental allergic rhinitis *via* the TLR4/NF-κB pathway. Int Immunopharmacol. (2020) 86:106522. doi: 10.1016/j.intimp.2020.106522 32585604

[114] Ness S Lin S Gordon JR. Regulatory dendritic cells, T cell tolerance, and dendritic cell therapy for immunologic disease. Front Immunol (2021) 12:633436. doi: 10.3389/fimmu.2021.633436 33777019 PMC7988082

